# The Vaginal Microbiome: Associations with Vaginal pH, Menopause and Metabolic Parameters

**DOI:** 10.3390/microorganisms13061317

**Published:** 2025-06-05

**Authors:** Yi-Chun Chen, Yi-Fen Chiang, Ko-Chieh Huang, Kai-Lee Wang, Yun-Ju Huang, Tzong-Ming Shieh, Mohamed Ali, Shih-Min Hsia

**Affiliations:** 1 School of Nutrition and Health Sciences, College of Nutrition, Taipei Medical University, Taipei 110301, Taiwan; 2 Department of Family and Community Medicine, Cheng Hsin General Hospital, Taipei 112401, Taiwan; 3 Department of Biotechnology and Food Technology, Southern Taiwan University of Science and Technology, Tainan City 710301, Taiwan; 4 School of Dentistry, College of Dentistry, China Medical University, Taichung 404333, Taiwan; 5 Institute of Oral Biology, College of Dentistry, National Yang Ming Chiao Tung University, Taipei 11221, Taiwan; 6 Department of Obstetrics and Gynecology, University of Chicago, Chicago, IL 60637, USA; 7 Clinical Pharmacy Department, Faculty of Pharmacy, Ain Shams University, Cairo 11566, Egypt; 8 School of Food Safety, Taipei Medical University, Taipei 110301, Taiwan; 9 Nutrition Research Center, Taipei Medical University Hospital, Taipei 110301, Taiwan; 10 Graduate Institute of Metabolism and Obesity Sciences, College of Nutrition, Taipei Medical University, Taipei 110301, Taiwan; 11 TMU Research Center for Digestive Medicine, Taipei Medical University, Taipei 110301, Taiwan

**Keywords:** vaginal microbiota, menopause, vaginal pH, community state types (CSTs), 16S rRNA sequencing, metabolic parameters, SGLT2 inhibitors, *Lactobacillus*

## Abstract

The vaginal microbiota, a critical determinant of women’s health, is influenced by hormonal and metabolic parameters across the lifespan. While *Lactobacillus* species are beneficial markers of vaginal health, microbial composition undergoes pronounced alterations after menopause. This study aimed to elucidate the associations between vaginal microbiota composition, vaginal pH, menopausal status, and metabolic parameters in Asian women. Vaginal secretion samples were collected from 40 women (20 premenopausal, 20 postmenopausal). Full-length 16S rRNA gene sequencing was used to characterize the microbiota, categorized into Community State Types (CSTs): CST-I + II (*Lactobacillus crispatus*/*gasseri*, protective), CST-III (*Lactobacillus iners*, neutral), and CST-IV (anaerobic bacteria, harmful). Vaginal pH and clinical data were assessed in relation to microbial profiles. CST distribution differed significantly by menopausal status and vaginal pH. Harmful-type CST-IV was more prevalent in postmenopausal women (70% vs. 40%, *p* < 0.05), while CST-III was dominant in premenopausal women (45% vs. 5%). CST-IV was associated with elevated pH (median 6.00, *p* = 0.026) and increased abundance of anaerobes including *Bacteroides*, *Fusobacterium*, *Porphyromonas*, *Prevotella*, and *Streptococcus*. Oral antibiotic use reduced both beneficial and harmful CSTs, shifting toward neutral CST-III (75%, *p* = 0.048). Use of sodium–glucose cotransporter-2 (SGLT2) inhibitors in postmenopausal women was associated with a higher prevalence of protective CST-I + II (57.14% vs. 8.33%, *p* < 0.05), though no significant impact on pathogen presence was observed. This study highlights the dynamic interplay between menopausal status, metabolic interventions, and vaginal microbiota composition. Findings may inform targeted strategies to maintain vaginal health in aging populations.

## 1. Introduction

The human body is colonized by a diverse array of symbiotic, commensal, and pathogenic microbes, collectively referred to as the microbiota, which includes bacteria, archaea, and fungi. When these microorganisms inhabit specific environments, they are described as the microbiota, whereas the term microbiome encompasses not only the microorganisms but also their genomes and the ecological interactions with their host and surrounding habitat [1].

The human microbiome, defined as the complete collection of microorganisms residing on and within the human body, plays a pivotal role in host physiology, immune system development, digestion, and detoxification processes [1]. The metagenome of the bacterial community alone contains at least 100 times more genes than the human genome, highlighting its critical role in maintaining homeostasis or contributing to the pathogenesis of various diseases [2]. While most of the research on the human microbiome over the past two decades has focused on colonic bacteria, uncovering significant associations with systemic physiological and pathological processes, other body sites, such as the skin, oral cavity, urinary tract, and reproductive tract, also harbor distinct microbial communities. These microbiota differ significantly from the gut microbiota, and even tissues previously considered sterile, such as the bladder, prostate, uterus, fallopian tubes, and ovaries, have been found to contain low-abundance microbial populations [2,3].

Unlike most body sites where high microbial diversity is often associated with health, the vaginal microbiota of women of reproductive age is characterized by relatively low diversity, typically dominated by a single *Lactobacillus* species. This has long been considered a hallmark of vaginal health, though its composition varies throughout a woman’s life stages, including childhood, puberty, pregnancy, and postmenopausal stages [4].

During the postmenopausal stage, declining estrogen leads to reduced glycogen, thinning of the vaginal epithelium, and increased vaginal pH. These changes disrupt the *Lactobacillus*-dominated microbiota, allowing diverse anaerobic bacteria such as *Gardnerella*, *Prevotella*, and *Bacteroides* to proliferate. These microbes secrete enzymes that degrade the protective mucus layer, triggering inflammation and a rise in vaginal symptoms. When these symptoms—discharge, odor, elevated pH, and clue cells—co-occur, they meet the clinical criteria for bacterial vaginosis (BV) based on Amsel’s criteria [5]. This menopausal microbiome shift increases susceptibility to recurrent infections and urogenital discomfort, highlighting the importance of understanding and managing these changes for women’s health [6].

Ravel et al. (2011) [7] introduced the concept of community state types (CSTs) based on 16S rDNA sequencing and analysis, classifying vaginal microbiota into five CSTs: CST-I (dominated by *Lactobacillus crispatus*), CST-II (*Lactobacillus gasseri*), CST-III (*Lactobacillus* *iners*), CST-IV (low *Lactobacillus* abundance with a mixture of anaerobes such as *Gardnerella*, *Atopobium*, and *Prevotella*), and CST-V (*Lactobacillus jensenii*). Among these, CST-IV is often associated with higher vaginal pH (pH > 4.5), biofilm formation, and bacterial vaginosis, whereas CST-I, II, and V, characterized by lower pH (<4.4), are considered protective. CST-III exhibits intermediate properties due to insufficient lactic acid production by *Lactobacillus iners*, making it highly dependent on host nutrients and susceptible to environmental changes, potentially leading to either protective or harmful effects on the host [7,8,9,10].

The aim of this study is to explore a clinically applicable approach to vaginal microbiome classification by incorporating both established community state types (CSTs) and identification of specific pathogenic taxa. Acknowledging the limitations of CST-based interpretation, we seek to enhance clinical relevance through pathogen-level insights. This study also examines associations between microbiota composition and host factors—including vaginal pH, menopausal status, metabolic indicators, and medication use—based on data from an underrepresented Asian population, with the goal of supporting future personalized strategies in women’s health.

## 2. Materials and Methods

### 2.1. Inclusion and Exclusion Criteria

The inclusion criteria for this study were female participants with a history of sexual activity and aged 30 years or older. Exclusion criteria included being pregnant or having undergone total hysterectomy. Written informed consent was obtained from all participants before enrollment. Menopause is diagnosed clinically following 12 consecutive months of amenorrhoea, provided that the patient is not using hormonal contraception [11].

This study was approved by the Institutional Review Board of Cheng Hsin General Hospital (CHGH-IRB, Approval No. (803)109A-42, NCT 06897800) and conducted in accordance with the Declaration of Helsinki.

The research protocol was reviewed and approved, and informed consent was obtained from all participants. This study was conducted in the outpatient department of Family Medicine at Cheng Hsin General Hospital, Taipei, Taiwan. The recruitment period lasted from 8 September 2022, to 18 August 2023.

### 2.2. Data Collection

#### 2.2.1. Physiological Measurements, Questionnaire Collection, and Biochemical Tests

Upon providing consent, participants underwent physiological measurements, including height, weight, waist circumference, body fat percentage, and blood pressure. Background information was collected using a structured questionnaire covering past medical history, current medication use, smoking and alcohol consumption frequency, and menstrual status. Vaginal antibiotic users were defined as participants who had received vaginal antibiotic suppository treatments within the past three months, and oral antibiotic users were defined as participants who had completed a course of oral antibiotics lasting more than five days within the past month. Participants underwent urinalysis and blood biochemical tests after fasting for at least eight hours. Blood tests included complete blood count (CBC), fasting blood glucose, glycated hemoglobin (HbA1c), and vitamin D levels.

Whole blood samples were obtained via venipuncture and collected into EDTA-treated vacutainers. For hematological assessment, complete blood count (CBC) was analyzed using an automated hematology analyzer. Fasting blood glucose (FBG) and HbA1c were measured using a standard enzymatic method and high-performance liquid chromatography (HPLC), respectively. Total circulating 25(OH)D levels were measured using a radioimmunoassay.

#### 2.2.2. Vaginal Secretion Sample Collection and Analysis

With informed consent, vaginal secretion samples were collected. Sampling was performed during the non-menstrual phase to ensure consistency in microbial analysis, and participants were instructed to abstain from sexual intercourse, vaginal douching, bathing, or the use of vaginal medications for at least 24 h before the test. Sampling was performed by a qualified medical doctor in a private and enclosed space using disposable vaginal speculums, pH test strips, and specific microbial collection tubes.

Vaginal pH was measured using pH test strips (pH-Fix 4.5–10.0, Macherey-Nagel, Düren, Germany), which contain fixed indicators for accurate pH determination. A sterile swab was used to collect vaginal fluid from the lateral vaginal wall, and the sample was then transferred onto the test strip. The pH value was determined by comparing the color change on the strip to the manufacturer’s standardized color chart. The measurement was performed under consistent lighting conditions to ensure accuracy.

Vaginal samples were collected using a genital tract sampling kit (Cat. No. B-MCK-BT-V01, Biotools, Madrid, Spain). The kit contains a DNA preservation solution, which stabilizes microbial DNA during transport and storage. Following sample collection, swabs were immediately placed into the provided preservation solution and stored at ambient temperature or refrigerated at 4 °C until further processing. Samples were transported under controlled conditions to maintain sample integrity. All samples were processed within 5 days of collection to minimize potential DNA degradation. The study employed third-generation sequencing technology to comprehensively sequence the complete 16S ribosomal RNA genes. This method utilizes all variable regions for high-resolution microbial species analysis.

### 2.3. Common Vaginal Pathogens Identification

A review of six studies published in the past six years identified 33 common vaginal pathogens [2,3,12,13,14,15]. Pathogens and their association with vaginal pH, menstrual status, and glycemic control medications are analyzed.

### 2.4. Full-Length 16S rRNA Gene Sequencing and Library Preparation

The full-length 16S ribosomal RNA gene sequencing process involved several key steps. Genomic DNA was extracted using the QIAamp PowerFecal Pro DNA Kit (Cat. No. 51804, QIAGEN GmbH, Hilden, Germany). Vaginal swab samples were collected and preserved using DNA/RNA Shield™ Collection Tubes with Swab (1 mL fill, Cat. No. R1107, Lot No. 217591, Zymo Research Corp., Irvine, CA, USA).

The 16S rRNA gene was amplified using barcoded universal primers (27F + 1492R) through Polymerase Chain Reaction (PCR), according to protocols provided by Pacific Biosciences of California, Inc. (PacBio, Menlo Park, CA, USA). The amplified DNA underwent quality control and optional pooling, followed by SMRTbell library preparation using the Sequel II Binding Kit 3.1 (Cat. No. 101-685-400, PacBio). Libraries were purified with AMPure PB Beads (PacBio), subjected to DNA damage repair, and ligated with sequencing adapters.

Sequencing was performed on the Sequel IIe System (PacBio, Menlo Park, CA, USA) to generate high-accuracy, full-length 16S rRNA gene sequences.

### 2.5. Bioinformatics Analysis

#### 2.5.1. Sequence Processing

Raw sequences (polymerase reads) were quality controlled. Subreads overlapping more than three times were used to generate Consensus Reads (CCS), improving sequence accuracy to HiFi reads (Read Quality > 30) with a 99.9% accuracy rate.

#### 2.5.2. Microbiome Analysis

HiFi reads were processed with DADA2 for ASV generation, including quality control, dereplication, chimera removal, and sequence aggregation. ASVs were annotated against reference databases (e.g., NCBI, GreenGenes, SILVA, eHOMD, UNITE) to generate species information and an ASVs Table. Further analyses based on the ASVs Table included alpha diversity analysis, species composition analysis, correlation analysis, phylogenetic analysis, and statistical testing analysis.

### 2.6. Statistics Analysis

The data analysis began with Community State Type (CST) classification, which was performed based on the microbial composition of each participant. Following CST classification, stratified analysis was conducted according to menopausal status to identify potential associations. Descriptive statistics were employed to summarize the data, including counts, percentages, medians, and interquartile ranges. For variable comparisons, continuous variables were analyzed using Kruskal–Wallis and Mann–Whitney U tests, while categorical variables were examined using Chi-squared or Fisher’s exact tests, with Bonferroni correction applied for post hoc comparisons. Diversity analysis included both alpha and beta diversity assessments. Alpha diversity was quantified using indices such as observed richness and Shannon diversity index, with boxplots used to visualize medians, dispersion, extremes, and outliers across groups. Statistical significance in alpha diversity comparisons was determined using the Wilcoxon Test.

## 3. Results

### 3.1. Distribution Among Community State Types (CST)

We analyzed key variables, including age, body mass index (BMI), smoking status, diabetes mellitus (DM), and community state types (CSTs), to compare postmenopausal and premenopausal groups (Table 1).

The mean age of participants was 51 years (SD = 8), with postmenopausal individuals significantly older (58 years, SD = 6) than premenopausal ones (43 years, SD = 7). The overall BMI averaged 25 (SD = 4), with no significant differences between groups. Smoking status showed no significant variation (*p* = 0.298). However, diabetes prevalence was notably higher in postmenopausal participants (60%) compared to premenopausal individuals (10%).

CST distribution varied significantly between the two groups. Protective types of CST I + II were observed in 20% of participants, with a slightly higher prevalence in postmenopausal (25%) than in premenopausal (15%) individuals. CST III, associated with neutral bacteria, was predominantly found in premenopausal individuals (45%) but was uncommon in the postmenopausal group (5%). In contrast, CST IV, characterized by harmful bacterial communities, was more prevalent among postmenopausal participants (70%) than in premenopausal individuals (40%). Chi-square analysis revealed a significant association between CST distribution and menopausal status (χ^2^ = 8.536, *p* = 0.014) (Table 1).

To further explore the clinical implications of CST differences, we compared vaginal symptoms and the presence of aerobic vaginitis across menopausal groups. As Table S1 summarizes the comparison of vaginal symptoms over three months between postmenopausal and premenopausal women, categorized by CST types and pathogen status. Meanwhile, no statistically significant associations were observed between CSTs and symptom presence (*p* > 0.05).

Furthermore, Table S2 details the comparison of aerobic vaginitis (AV) presence across CSTs. Although the overall association was not statistically significant (*p* = 0.319), CST IV was more frequently observed among individuals with AV, particularly in the postmenopausal group. These findings suggest a potential link between vaginal dysbiosis and clinical conditions such as AV, especially in postmenopausal women.

Microbial classification identified seven samples belonging to CST I, characterized by *Lactobacillus crispatus*, and one sample classified as CST II, dominated by *Lactobacillus gasseri.* Ten samples were assigned to CST III, dominated by *Lactobacillus iners*, while 22 samples were categorized as CST IV due to a depletion or low abundance of *Lactobacillus* species (Table 2). We also examined the relationship between CSTs and the presence of pathogenic bacteria (Table S3). Symptomatic participants with CST IV were more likely to harbor pathogens such as Group B *Streptococcus* and *Staphylococcus aureus*. Conversely, CST IV was also observed among asymptomatic individuals without detectable pathogens (Table S4), indicating that a dysbiotic microbiome may exist even in the absence of overt infection.

**Figure 1:**
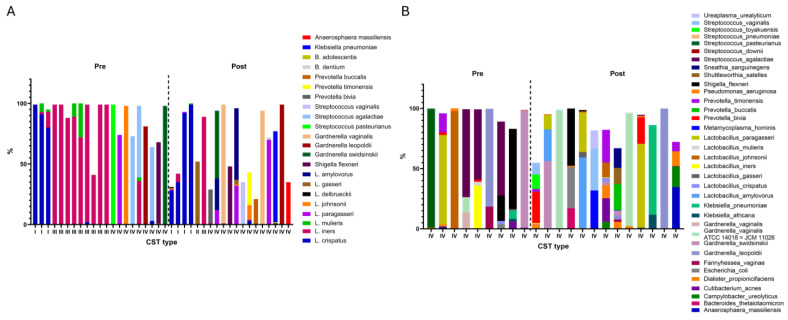
Microbiome composition of participants by menopausal status and Community State Types (CSTs). (**A**) Relative abundances of *Lactobacillus* species in the vaginal microbiota of premenopausal (Pre) and postmenopausal (Post) participants. (**B**) Relative abundances of vaginal microbiota species in participants classified as CST IV.

Figure 1A illustrates the distribution of *Lactobacillus* species across premenopausal and postmenopausal groups. In five samples, both *Lactobacillus crispatus* and *Lactobacillus iners* were present simultaneously; these were classified as CST I, III, or IV depending on their relative abundance (Table 2). Samples lacking dominance by *L. crispatus*, *L. gasseri*, or *L. iners*, where their combined relative abundance was less than 10%, were designated as CST IV.

Collectively, these findings highlight a significant shift in vaginal microbiota composition with menopausal status, particularly the increased prevalence of CST IV in postmenopausal women, which may be associated with a greater risk of vaginal dysbiosis and clinical symptoms such as aerobic vaginitis.

### 3.2. Community State Types (CST) Classification

#### 3.2.1. Vaginal pH

Vaginal pH levels exhibited a statistically significant difference among CST groups, as determined by a Kruskal–Wallis non-parametric one-way ANOVA (*p* = 0.026). Subsequent post hoc analysis using the Bonferroni correction revealed that the vaginal pH in CST IV was significantly higher than that in CST III. CST IV exhibited the highest median vaginal pH (6.00), significantly higher than CST III (4.50), and CST I + II (5.00) as determined by post hoc analysis (Table 3).

#### 3.2.2. Menopausal Status

The proportion of postmenopausal participants varied significantly among CST groups (*p* = 0.014). CST IV had the highest prevalence of postmenopausal participants (70%), followed by CST I + II (50%), and CST III (10%) (Table 3).

#### 3.2.3. Use of Oral Antibiotics

Oral antibiotic use was significantly associated with CST classification (*p* = 0.048). Participants who used antibiotics had lower proportions of both protective (CST I + II) and harmful bacteria (CST IV), while exhibiting a markedly higher proportion of neutral bacteria (CST III) (75%). Other variables, including BMI, glucose levels, HbA1c, body fat percentages, vitamin D level, and the use of SGLT2 inhibitors, did not exhibit statistically significant differences across CST groups (all *p* > 0.05) (Table 3).

Overall, the findings indicate significant associations between vaginal pH, menopausal status, antibiotic use, and CST classification. CST IV was associated with higher vaginal pH, a higher prevalence of postmenopausal individuals, and a lower proportion of protective bacteria in those using oral antibiotics.

### 3.3. Postmenopausal Group and Premenopausal Group

**Figure 2:**
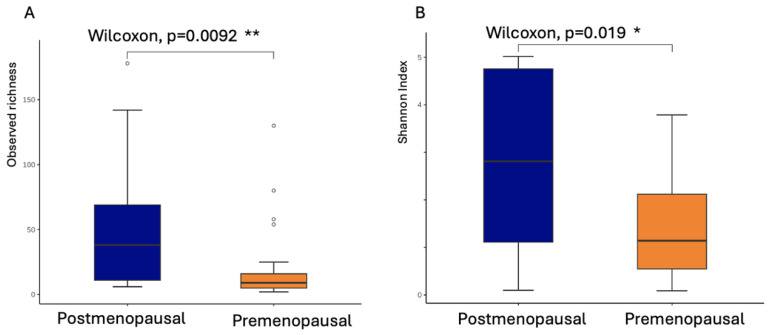
Vaginal microbial richness and diversity in postmenopausal women. (**A**) Observed richness indices and (**B**) Shannon diversity index in the postmenopausal group and premenopausal group. Statistical significance was assessed using the Wilcoxon rank-sum test (*p* = 0.0092 for observed richness; *p* = 0.019 for Shannon index). * *p* < 0.05, ** *p* < 0.01.

Table 4 presents the univariate analysis of factors influencing CST classification within the postmenopausal group. The results indicate that postmenopausal women using SGLT2 inhibitors had a higher proportion of protective bacteria (CST I + II) compared to harmful bacteria (CST IV) (57.14% vs. 42.68%). In contrast, among postmenopausal women not using SGLT2 inhibitors, the proportion of protective bacteria was markedly lower (8.33%), while harmful bacteria predominated (91.67%). The postmenopausal group exhibited significantly higher bacterial richness and diversity, as measured by observed richness indices (Wilcoxon, *p* = 0.0092) and Shannon diversity index (Wilcoxon, *p* = 0.019), both of which reached statistical significance (Figure 2A,B).

In contrast, Table 5 summarizes the univariate analysis of CST classification within the premenopausal group, where no significant associations were observed.

### 3.4. Pathogens and Their Association with Vaginal pH, Menstrual Status, and Glycemic Control Medications

A total of 26 pathogens were identified among 40 participants (Table 6). The analysis focused on the presence and abundance of these pathogens in relation to vaginal pH, menopausal status, and the use of SGLT2 inhibitors for glycemic control.

Regarding vaginal pH levels, five pathogens *Bacteroides*, *Fusobacterium*, *Porphyromonas*, *Prevotella*, and *Streptococcus* exhibited a significant positive correlation between their presence and abundance with vaginal pH. In the postmenopausal group, Ureaplasma showed a significant negative correlation with vaginal pH presence. In the premenopausal group, three pathogens (*Actinomyces*, *Fusobacterium*, and *Streptococcus*) were positively associated with vaginal pH.

When comparing menstrual status, three pathogens *Dialister*, *Porphyromonas*, and *Peptoniphilus* demonstrated significantly higher presence or abundance in the postmenopausal group compared to the premenopausal group. However, no significant associations were identified between specific pathogens and the use of SGLT2 inhibitors, indicating that glycemic control via this medication did not influence pathogen profiles in the study population.

## 4. Discussion

Our study first revealed that among Asian women, CST III was the most prevalent vaginal microbiome type during the reproductive stage, whereas CST IV predominated in the menopausal stage. A 2011 report comparing CST distribution across different ethnic groups among reproductive-age women [7] found that CST III was more common in Asians, CST I predominated among Caucasians, and CST IV was more prevalent in Black populations. Furthermore, a 2014 study focusing on a predominantly Caucasian population (80%) reported an increase in CST III prevalence during the peri-menopause stage [16]. These findings underscore the significant role of menopausal status in shaping vaginal microbial profiles. Our study extends these findings by providing specific data on postmenopausal Asian women, demonstrating a notable rise in CST IV prevalence to 70% in this group compared to 40% among reproductive-age women, a statistically significant difference.

The findings of this study revealed that vaginal pH, menopausal status, and oral antibiotic use significantly influence the distribution of vaginal bacterial Community State Types (CSTs). These findings underscore the intricate interplay between host physiological factors and external interventions in determining CST dynamics and highlight the need for targeted approaches in managing vaginal health across different life stages.

### 4.1. Vaginal pH Level

The human vaginal microbiota is dominated by Lactobacillus, which maintains a protective acidic environment, with pH lowest in the vagina and increasing toward the uterus [17]. The human vagina is uniquely dominated by Lactobacillus (>70%) and maintains a lower pH (~4.5) than other mammals, where Lactobacillus levels are typically <1% and vaginal pH ranges from 5.4 to 7.8 [18].

In this study, we found that the average vaginal pH among participants ranged from 4.5 to 6.0. Although higher than some reported values in the literature, this is consistent with other findings, with the highest pH values observed in CST-IV groups [8]. A 2022 study reported that CST-IV microbiota are characterized by a pH above 4.5, which promotes the formation of polymicrobial biofilms and increases susceptibility to bacterial vaginosis. In contrast, CST-I and CST-II types, with pH values below 4.4, provide protective effects for the vaginal environment. CST-III, dominated by *Lactobacillus iners*, represents an intermediate profile. However, *Lactobacillus iners*, with its insufficient lactate production and reliance on host nutrients, is highly sensitive to environmental changes and may neither protect the host nor potentially contribute to host harm.

### 4.2. Menopausal Status

A distinct bacterial community state (CST IV), characterized by a low relative abundance of *Lactobacillus*, has been associated with vulvovaginal atrophy (VVA) [16]. A 2014 study, predominantly involving a Caucasian population (over 75%), similarly reported a higher prevalence of CST IV among postmenopausal women and those with vaginal atrophy. Post menopause induces several changes in the vaginal environment, including reductions in estrogen and glycogen levels, thinning of the vaginal epithelium to a pre-pubertal-like state, a shift in vaginal microbiota from *Lactobacilli* dominance to increased microbial diversity (CST IV), elevated vaginal pH, diminished secretions, dryness, and dyspareunia. Our study contributes novel data on Asian women, corroborating the pivotal role of menopause in altering vaginal microbiota. Notably, we observed an exceptionally high prevalence of CST IV, reaching 70%, in this population. This highlights the consistent influence of menopause on vaginal microbiota composition across diverse ethnic groups.

### 4.3. Antibiotics

A 2015 study found that antibiotics rapidly cleared anaerobes within 3 days; *Lactobacillus jensenii* regrew fastest, *Lactobacillus iners* increased gradually, while *Lactobacillus crispatus* remained stable [19]. Although our cross-sectional study did not involve comparisons before and after treatment, our findings align with this report. Within our antibiotic-treated group, *Lactobacillus iners* (CST III) constituted up to 75% of the microbiota, as CST IV accounted for 25%. In contrast, the proportion of pathogenic bacteria in the group not receiving oral antibiotic treatment was 58.3%. This observation supports the hypothesis that antibiotics treatment results in a transient microbial vacuum, which is subsequently filled by the growth of *Lactobacillus iners*.

### 4.4. Diabetes Mellitus

In our study, no significant association was observed between Type 2 diabetes status, HbA1c levels, and the CST classification of vaginal microbiota in both postmenopausal and premenopausal women. To date, limited research has explored the relationship between glucose metabolism and vaginal microbiota, with most studies focusing on gestational diabetes mellitus (GDM) in reproductive-age women [20].

Studies on type 2 diabetes and vaginal microbiota remain limited, often using smear or culture methods. Recent data show reduced Lactobacillus iners and higher prevalence of Candida, Leptothrix, and Gardnerella in diabetic women [21,22].

It is worth noting that SGLT2 inhibitors (sodium–glucose cotransporter 2 inhibitors), a class of glucose-lowering medications, work by promoting glucose excretion through the kidneys and urine. This mechanism is often associated with side effects such as discomfort in the genitourinary tract due to increased sugar concentrations in these regions. A 2023 study using data from China’s FDA Adverse Event Reporting System (FAERS) highlighted a significant association between SGLT2 inhibitors and urinary/genital mucosal infections [21]. Additionally, a report from Japan found that SGLT2 treatment increased the culture-colony counts of pathogenic bacteria [22]. However, this study did not provide information on changes in lactobacilli populations.

In our study, postmenopausal women using SGLT2 inhibitors exhibited a significantly higher prevalence of beneficial vaginal microbiota classified as CST I + II compared to non-users. While the current evidence does not fully explain this association, one plausible hypothesis is that elevated sugar levels in the vaginal environment may serve as an energy source for beneficial lactobacilli growth. This potential link between sugar and lactobacilli also aligns with findings in dental caries research. Studies have shown that the colonization of lactobacilli in three human sites—carious lesions, the stomach, and the vagina—requires key conditions, including a retentive anaerobic environment, low pH, and an ample supply of carbohydrates [23].

### 4.5. Vitamin D

In recent years, vitamin D has been extensively studied across various medical fields. However, research specifically focusing on the relationship between vitamin D and vaginal health has primarily concentrated on its association with HPV infection and menopausal vaginal atrophy [24,25,26]. More recently, meta-analyses have highlighted a potential link between vitamin D deficiency and bacterial vaginosis (BV) [27,28]. Despite these associations, there is currently no conclusive evidence supporting the efficacy of vitamin D supplementation in the treatment of BV. A randomized controlled trial (RCT) conducted by Holm in 2017 investigated the adjunctive use of oral metronidazole combined with nine doses of 50,000 IU of cholecalciferol over 24 weeks, yet no significant improvement was observed compared to the control group [29].

Although the clinical efficacy of vitamin D in vaginal infections remains unconfirmed, emerging research has begun to explore its potential role in shaping the vaginal microbiome, aiming to better understand the underlying physiological and pathological mechanisms. A 2019 study demonstrated a correlation between specific vaginal microbial taxa and serum 25(OH)D concentrations, revealing a negative correlation between *Megasphaera* abundance and serum 25(OH)D levels in African-American women (*p* = 0.0187). Conversely, in the European control group, serum 25(OH)D levels were positively correlated with the abundance of *Lactobacillus crispatus* [30].

In contrast to these findings, our study did not identify a significant association between vitamin D concentration and vaginal microbial community state types (CSTs). These results suggest that while vitamin D may play a role in vaginal microbiome composition, its influence may be modulated by host-specific factors such as ethnicity, genetic predisposition, and environmental influences. Further investigations, particularly well-designed longitudinal and interventional studies, are warranted to clarify the causal relationship between vitamin D levels and vaginal microbiome dynamics, as well as to explore potential therapeutic implications.

### 4.6. Diversity in Vaginal Microbiota

As summarized in previous research [31], our study similarly found that vaginal microbiota diversity increased in postmenopausal women, as confirmed through Shannon and Simpson index analyses. This was accompanied by higher microbial richness. Increased vaginal microbial diversity has also been observed in patients with bacterial vaginosis [32] and in those experiencing vaginal dryness [33], further supporting the link between altered vaginal conditions and shifts in microbial composition.

### 4.7. Specific Pathogens

Based on a synthesis of six recent studies (2019–2022) [2,3,12,13,14,15], 33 common vaginal pathogenic bacterial genera were identified. From these, 26 genera present in our study population were selected for further analysis, comparing their associations with vaginal pH, menstrual status, and diabetes medication use. Our findings revealed five genera *Bacteroides*, *Fusobacterium*, *Porphyromonas*, *Prevotella*, *Streptococcus* with a significant positive correlation with vaginal pH, indicating that higher vaginal alkalinity increases the likelihood of detecting these bacteria. Additionally, in premenopausal women, the associations between vaginal pH and *Fusobacterium*, *Actinomyces,* and *Streptococcus* was more pronounced. Regarding menstrual status, our analysis showed that three genera—*Dialister*, *Porphyromonas*, *Peptoniphilus*—were significantly more abundant in postmenopausal women. These findings align closely with a 2014 report, which identified *Peptoniphilus*, *Prevotella*, and *Streptococcus* as prevalent genera in postmenopausal women experiencing vaginal atrophy (VVA) symptoms, demonstrating a high degree of consistency between our results and previous studies.

These findings emphasize the complex interplay between vaginal pathogens and environmental factors such as vaginal pH, menstrual status, and pharmacological interventions. Vaginal pH appears to play a significant role in shaping the presence and abundance of specific pathogens, with notable differences observed between postmenopausal and premenopausal participants. In contrast, the use of SGLT2 inhibitors was not associated with significant changes in pathogen profiles, suggesting that glycemic control medications may have limited influence on vaginal microbiota in this study. This analysis contributes to a better understanding of how environmental and physiological factors influence the distribution of vaginal pathogens, providing insights into future research and potential clinical interventions.

This study is limited by its small sample size, which may affect the generalizability of the findings. Moreover, it examines only the bacterial component of the vaginal microbiome through 16S rRNA sequencing, without assessing fungal or protozoal communities, thereby narrowing the scope of microbial evaluation. Additionally, while follicle-stimulating hormone and estrogen level measurements are not essential for defining menopausal status, their inclusion could provide stronger evidence or offer additional perspectives. The lack of detailed documentation on antibiotic types, indications, and probiotic use also limits analysis of their impact on vaginal microbiota composition. The cross-sectional design further limits the ability to evaluate causal relationships or temporal dynamics, highlighting the need for future research to adopt a cohort study design for longitudinal analysis. Although this study utilized TGS 16S technology, enabling analysis down to the species level, the current sample size is more suited for statistical analysis at the genus level. With an increased sample size in future studies, analysis at the species level can be conducted to offer more detailed and refined insights.

## 5. Conclusions

This study explored the dynamics of the vaginal microbiome across various physiological, metabolic, and clinical contexts, highlighting notable associations between vaginal pH levels, bacterial community state types (CSTs), and influencing factors.

Significant associations were observed between vaginal pH, menopausal status, and CST distribution. Harmful type bacteria CST IV, linked to higher pH and reduced Lactobacillus, was predominant in postmenopausal women versus premenopausal women, while neutral type CST III dominated in premenopausal women. Vaginal pH positively correlated with *Bacteroides*, *Fusobacterium*, *Porphyromonas*, *Prevotella*, and *Streptococcus*. Postmenopausal women had higher levels of *Dialister*, *Porphyromonas*, and *Peptoniphilus*. Oral antibiotics increased neutral type CST III but reduced protective type CST I + II and harmful type CST IV. SGLT2 inhibitors raised protective type CST I + II in postmenopausal women without altering pathogen profiles.

While these findings suggest potential patterns linking host factors and microbiome composition, the small sample size—particularly in subgroup analyses—warrants caution in interpretation. Future studies with larger cohorts are needed to validate these preliminary observations and better elucidate the clinical relevance of such associations. Overall, this study provides initial insight into the complex interplay between microbial composition and host physiology, contributing to our understanding of the vaginal microbiome’s potential role in health and disease.

## Figures and Tables

**Table 1:** The results of the sample grouping based on menopausal period.

	All (n = 40)	Postmenopausal (n = 20)	Premenopausal (n = 20)	$t/\chi^{2}$	*p*
Age, m (sd)	51(8)	58(6)	43(7)	7.068	<0.001 ***
BMI, m (sd)	25(4)	26(4)	24(4)	1.513	0.139
Smoke, n (%)				2.421	0.298
Never	29(73)	13(65)	16(80)		
Ex	2(5)	2(10)	0(0)		
Current	9(23)	5(25)	4(20)		
DM, n (%)				8.901	0.003 **
NO	26(65)	8(40)	18(90)		
Yes	14(35)	12(60)	2(10)		
CST, n (%)				8.536	0.014 *
I + II	8(20)	5(25)	3(15)		
III	10(25)	1(5)	9(45)		
IV	22(55)	14(70)	8(40)		

CST: Community State Types.; DM: Diabetes Mellitus; BMI: Body Mass Index. m(sd): mean ± standard deviation. * *p* < 0.05, ** *p* < 0.01, *** *p* < 0.001.

**Table 2:** Description of the microbial Composition and Community State Type (CST) classification.

CST	Microbiome
I	**99% *L. crispatus***
I	**91% *L. crispatus***, **2% *L. iners***, **7% *L. mulieris***
I	**28% *L. crispatus***, **1% *L. paragasseri***, **1% *L. johnsonii***, **1% *L. delbrueckii***
I	**80% *L. crispatus*, 13% *L. iners***, **2% *L. mulieris***
I	**35% *L. crispatus*, 7% *L. iners***
I	**92% *L. crispatus***, **1% *L. paragasseri***
I	**99% *L. crispatus***, **1% *L. mulieris***
II	***52% L. gasseri***
III	**99% *L. iners***
III	**99% *L. iners***
III	**88% *L. iners***
III	**89% *L. iners***, **11% *L. mulieris***
III	**72% *L. iners***, **28% *L. mulieris***
III	**97% *L. iners***, **2% *L. crispatus***
III	**89% *L. iners***
III	**41% *L. iners***
III	**99% *L. iners***
III	**99% *L. iners***
IV	28% *Prevotella bivia*, **1% *L. iners***
IV	56% *Gardnerella swidsinskii*, **12% *L. paragasseri***, **26% *L. amylovorus***
IV	98% *Gardnerella vaginalis*, **1% *L. iners***
IV	99% *Streptococcus pasteurianus*
IV	48% *Shigella flexneri*
IV	**59% *L. amylovorus***, **4% *L. gasseri***, **32% *L. paragasseri*, 1% *L.* johnsonii**
IV	**74% *L. paragasseri***
IV	35% *Streptococcus vaginalis*
IV	27% *Prevotella timonensis*, **3% *L. crispatus***, **1% *L. iners***, **12% *L. johnsonii***
IV	21% *Prevotella buccalis*
IV	94% *Gardnerella vaginalis*
IV	**70% *L. paragasseri***, **2% *B. dentium***
IV	**98% *L. johnsonii***
IV	73% *Streptococcus agalactiae*
IV	75% *Klebsiella pneumoniae*, **1% *L. amylovorus***, **1% *B. adolescentis***
IV	59% *Streptococcus agalactiae*, **36% *L. iners***, **3% *L. mulieris***
IV	81% *Gardnerella leopoldii*
IV	61% *Streptococcus agalactiae*, **3% *L. crispatus***
IV	67% *Shigella flexneri*, **1% *L. gasseri***
IV	99% *Gardnerella leopoldii*
IV	98% *Gardnerella swidsinskii*
IV	35% *Anaerosphaera massiliensis*

**Table 3:** Univariate analysis of factors across different bacterial Community State Types (CSTs).

	CST I + CST II (n = 8)		CST III (n = 10)		CST IV (n = 22)		*p* ^a^	Post Hoc ^b^
	Median/n	IQR/%	Median/n	IQR/%	Median/n	IQR/%		
Vaginal tract pH	5.00	1.88	4.50	1.00	6.00	1.63	0.026	III < IV
HbA1c	5.55	2.13	5.65	1.05	5.65	1.13	0.612	
Glucose	98.00	32.00	95.50	13.75	92.00	30.00	0.737	
BMI	24.35	5.56	25.99	7.93	24.00	6.05	0.684	
Vitamin D (25(OH)D)	21.65	16.70	19.80	8.10	20.95	8.35	0.362	
Waist	87.25	14.25	85.75	15.13	81.00	11.63	0.244	
Average Body Fat	35.20	5.30	34.90	16.13	34.25	8.93	0.793	
Trunk Fat	34.80	6.32	33.75	19.43	34.80	11.20	0.811	
Visceral Fat	8.00	5.00	7.00	7.50	7.00	4.25	0.771	
Menopause							0.014	III, IV
Postmenopause	5	25.00	1	5.00	14	70.00		
Premenopause	3	15.00	9	45.00	8	40.00		
Smoking history							0.972	
No	6	20.69	7	24.14	16	55.17		
Yes	2	18.18	3	27.27	6	54.55		
BMI							0.492	
Normal	2	11.76	4	23.53	11	64.71		
Overweight	3	33.33	1	11.11	5	55.56		
Obese	3	21.43	5	35.71	6	42.86		
Vitamin D							0.158	
<20	2	10.50	7	36.8	10	52.60		
≥20	6	28.60	3	14.30	12	57.10		
HbA1c							0.121	
<6.5	5	16.10	10	32.30	16	51.60		
≥6.5	3	33.30	0	0.00	6	66.70		
OAD							0.400	
No	4	14.80	8	29.60	15	55.60		
Yes	4	30.80	2	15.40	7	53.80		
SGLT2 inhibitors							0.100	
No	4	12.90	9	29.00	18	58.10		
Yes	4	44.40	1	11.10	4	44.40		
Antibiotics (Vaginal)							0.423	
No	8	21.10	10	26.30	20	52.60		
Yes	0	0.00	0	0.00	2	100.00		
Antibiotics (oral)							0.048	
No	8	22.20	7	19.40	21	58.30		
Yes	0	0.00	3	75.00	1	25.00		
DM							0.407	
No	4	15.38	8	30.77	14	53.85		
Yes	4	28.57	2	14.29	8	57.14		

^a^ Kruskal–Wallis test or Chi-squared test; ^b^ Bonferroni method. OAD: Oral Antidiabetic Drugs. DM: Diabetes Mellitus. SGLT2 inhibitors: Sodium–glucose co-transporter-2 inhibitors. BMI: Body Mass Index.

**Table 4:** Univariate analysis of factors among different bacterial Community State Types among the postmenopausal group.

	CST I + CST II (n = 5)		CST IV (n = 14)		*p* ^a^
	Median/n	IQR/%	Median/n	IQR/%	
Vaginal tract PH	6.00	1.75	6.00	1.50	0.391
HbA1c	7.10	2.25	6.05	1.22	0.754
Glucose	114.00	31.50	102.50	40.25	0.559
BMI	27.55	5.69	25.52	5.21	0.622
Vitamin D	20.80	13.00	21.90	10.13	0.559
Waist	94.00	11.75	85.00	11.38	0.056
Average Body Fat	36.30	8.05	37.55	10.70	0.893
Trunk Fat	36.10	10.50	37.75	12.98	0.964
Visceral Fat	8.00	5.00	9.00	7.00	0.893
Smoking history					0.999
No	3	25.00	9	75.00	
Yes	2	28.57	5	71.43	
BMI					0.405
Normal	0	0.00	4	100.00	
Overweight	2	33.33	4	66.67	
Obese	3	33.33	6	66.67	
Vitamin D					0.603
<20	1	14.29	6	85.71	
≥20	4	33.33	8	66.67	
HbA1c					0.603
<6.5	2	18.18	9	81.82	
≥6.5	3	37.50	5	62.50	
OAD					0.303
No	1	11.11	8	88.89	
Yes	4	40.00	6	60.00	
SGLT2 inhibitors					0.038
No	1	8.33	11	91.67	
Yes	4	57.14	3	42.86	
Antibiotics (Vaginal)					0.999
No	5	27.78	13	72.22	
Yes	0	0.00	1	100.00	
Antibiotics (oral)					0.999
No	5	27.78	13	72.22	
Yes	0	0.00	1	100.00	
DM					0.338
No	1	12.50	7	87.50	
Yes	4	36.36	7	63.64	

OAD: Oral Antidiabetic Drugs; DM: Diabetes Mellitus; SGLT2 inhibitors: Sodium–glucose co-transporter-2 inhibitors; BMI: Body Mass Index; IQR: Interquartile Range. ^a^: Kruskal-Wallis test or Chi-squared test.

**Table 5:** Univariate analysis of factors among different bacterial Community State Types among the premenopausal group.

	CST I + CST II (n = 3)		CST III (n = 9)		CST IV (n = 8)		*p* ^a^
	Median/n	IQR/%	Median/n	IQR/%	Median/n	IQR/%	
Vaginal pH	4.50	-	4.50	1.50	5.50	1.38	0.177
HbA1c	5.10	-	5.60	1.00	5.45	0.63	0.465
Glucose	88.00	-	95.00	11.50	85.00	11.25	0.160
BMI	22.31	-	27.42	7.71	21.90	2.16	0.051
Vitamin D	22.50	-	19.80	9.70	20.40	7.53	0.623
Waist	79.00	-	88.50	13.25	78.00	15.75	0.105
Average Body Fat	34.30	-	35.80	14.35	31.35	7.05	0.264
Trunk Fat	34.50	-	34.20	17.40	30.90	8.25	0.466
Visceral Fat	6.00	-	7.00	7.50	5.50	2.75	0.327
Smoking history							0.362
No	3	18.75	6	37.50	7	43.75	
Yes	0	0.00	3	75.00	1	25.00	
BMI							0.061
Normal	2	16.67	3	25.00	7	58.33	
Overweight	1	33.33	1	33.33	1	33.33	
Obese	0	0.00	5	100.00	0	0.00	
Vitamin D							0.564
<20	1	9.09	6	54.55	4	36.36	
≥20	2	22.22	3	33.33	4	44.44	
HbA1c							0.454
<6.5	3	15.79	9	47.37	7	36.84	
≥6.5	0	0.00	0	0.00	1	100.00	
OAD							0.818
No	3	16.67	8	44.44	7	38.89	
Yes	0	0.00	1	50.00	1	50.00	
SGLT2							0.818
No	3	16.67	8	44.44	7	38.89	
Yes	0	0.00	1	50.00	1	50.00	
Antibiotics (Vaginal)							0.454
No	3	15.79	9	47.37	7	36.84	
Yes	0	0.00	0	0.00	1	100.00	
Antibiotics (oral)							0.116
No	3	17.65	6	35.29	8	47.06	
Yes	0	0.00	3	100.00	0	0.00	
DM							0.818
No	3	16.67	8	44.44	7	39.89	
Yes	0	0.00	1	50.00	1	50.00	

OAD: Oral Antidiabetic Drugs; DM: Diabetes Mellitus; SGLT2 inhibitors: Sodium–glucose co-transporter-2 inhibitors; BMI: Body Mass Index; IQR: Interquartile Range. ^a^: Kruskal-Wallis test or Chi-squared test.

**Table 6:** Comparative analysis of pathogenic bacteria across different vaginal pH levels, menstrual states, and diabetes medication status.

Pathogen	n	Vaginal pH Level						Cases % Among					
		All Cases		Post-Menopause		Pre-Menopause		Post-Menopause (%) / Pre-Menopause (%)		SGLT2 (−) (%)/SGLT2 (+) (%)			
										Post-Menopause		Pre-Menopause	
		r	*p*	r	*p*	r	*p*	%/%	*p*	%/%	*p*	%/%	*p*
***Acinetobacter***	1							5.0/0.0		7.0/0.0		-	
positive		0.148	0.361	0.161	0.479	-	-		1.000		1.000		-
abundance													
***Actinomyces***	6							20.0/10.0		23.1/14.3		11.1/0.0	
positive		0.256	0.111	−0.099	0.679	0.531	0.016 *		0.658		1.000		1.000
abundance		0.231	0.151	−0.149	0.532	0.532	0.016 *		0.602		0.699		0.853
***Atopobium***	1							5.0/0.0		7.7/0.0		-	
positive		0.071	0.665	0.000	1.000	-	-		1.000		1.000		-
abundance													
***Anaerococcus***	14							45.0/25.0		38.5/57.1		27.8/0.0	
positive		0.090	0.580	−0.132	0.578	0.074	0.758		0.320		0.642		1.000
abundance		0.115	0.479	0.100	0.676	−0.094	0.693		0.221		0.757		0.674
***Bacteroides***	7							25.0/10.0		23.1/28.6		11.1/0.0	
positive		0.313	0.049 *	0.375	0.103	0.106	0.656		0.405		1.000		1.000
abundance		0.317 *	0.047 *	0.347	0.134	0.129	0.587		0.398		0.938		0.853
***Clostridium***	2							10.0/0.0		0.0/28.6		-	
positive		0.212	0.188	0.234	0.320	-	-		0.468		0.111		-
abundance													
***Dialister***	15							55.0/20.0		53.8/57.1		16.7/50.0	
positive		0.198	0.221	0.079	0.739	0.023	0.924		0.050		1.000		0.368
abundance		0.248	0.122	0.213	0.367		0.936		0.035 *				
***Escherichia***	2							5.0/5.0		7.7/0.0		5.6/0.0	
positive		0.101	0.535	0.302	0.195	0.042	0.861		1.000		1.000		1.000
abundance													
***Finegoldia***	11							40.0/15.0		46.2/28.6		16.7/0.0	
positive		0.274	0.087	0.045	0.851	0.293	0.210		0.157		0.642		1.000
abundance		0.247	0.124	0.106	0.656		0.283		0.114				
***Fusobacterium***	9							35.0/10.0		38.5/28.6		11.1/0.0	
positive		0.354 *	0.025 *	0.074	0.758	0.531 *	0.016 *		0.130		1.000		1.000
abundance		0.376 *	0.017 *	0.099	0.677		0.017 *		0.231				
***Gardnerella***	17							55.0/30.0		61.5/42.9		33.3/0.0	
positive		0.096	0.556	−0.371	0.108	0.447 *	0.048		0.201		0.642		1.000
abundance		0.094	0.566	−0.295	0.206		0.060		0.165				
***Gemella***	8							30.0/10.0		30.8/28.6		11.1/0.0	
positive		0.091	0.577	−0.230	0.329	0.273	0.244		0.235		1.000		1.000
abundance		0.092	0.570	−0.245	0.298		0.227		0.341				
***Leptotrichia***	4							15.0/5.0		15.4/14.3		5.6/0.0	
positive		0.044	0.787	−0.307	0.187	0.334	0.150		0.605		1.000		1.000
abundance		0.028	0.864	−0.320	0.169		0.150		0.565				
***Mycoplasma***	2							10.0/0.0		15.4/0.0		-	
positive		0.207	0.199	0.220	0.352	-	-		0.468		0.521		-
abundance													
***Megasphaera***	2							5.0/5.0		7.7/0.0		5.6/0.0	
positive		0.207	0.199	0.000	1.000	0.334	0.150		1.000		1.000		1.000
abundance													
***Mobiluncus***	4							15.0/5.0		15.4/14.3		5.6/0.0	
positive		0.118	0.470	0.283	0.227	−0.250	0.287		0.605		1.000		1.000
abundance		0.133	0.412	0.291	0.212		0.287		0.565				
***Porphyromonas***	13							50.0/15.0		53.8/42.9		16.7/0.0	
positive		0.454	0.003 *	0.421	0.064	0.293	0.210		0.043 *		1.000		1.000
abundance		0.417	0.007 *	0.339	0.143		0.218		0.060				
**Parvimonas**	3							10.0/5.0		7.7/14.3		5.6/0.0	
positive		0.213	0.186	0.000	1.000	0.334	0.150		1.000		1.000		1.000
abundance													
***Peptostreptococcus***	2							10.0/0.0		7.7/14.3		-	
positive		0.111	0.494	0.015	0.951	-	-		0.468		1.000		-
abundance													
***Peptococcus***	2							10.0/0.0		7.7/14.3		-	
positive		0.207	0.199	0.220	0.352	-	-		0.468		1.000		-
abundance													
***Peptoniphilus***	12							50.0/10.0		46.2/57.1		11.1/0.0	
positive		0.245	0.127	0.176	0.459	0.015	0.949		0.016 *		1.000		1.000
abundance		0.256	0.111	0.266	0.257		0.987		0.023 *				
***Prevotella***	20							65.0/35.0		69.2/57.1		33.4/50.0	
positive		0.379	0.016 *	0.138	0.562	0.372	0.106		0.114		0.651		1.000
abundance		0.332	0.036 *	0.140	0.556		0.201		0.056				
***Sneathia***	1							5.0/0.0		7.7/0.0		-	
positive		0.071	0.665	0.000	1.000	-	-		1.000		1.000		-
abundance													
***Shuttleworthia***	1							5.0/0.0		7.7/0.0		-	
positive		0.071	0.665	0.000	1.000	-	-		1.000		1.000		-
abundance													
***Streptococcus***	25							65.0/60.0		61.5/71.4		61.1/50.0	
positive		0.346	0.029 *	0.396	0.084	0.297	0.203		1.000		1.000		1.000
abundance		0.420	0.007 *	0.263	0.263		0.020 *		0.989				
***Ureaplasma***	11							35.0/20.0		38.5/28.6		16.7/50.0	
positive		−0.079	0.628	−0.497	0.026 *	0.216	0.360		0.479		1.000		0.368
abundance		−0.020	0.901	−0.405	0.077		0.264		0.398				

r: Spearman’s rank correlation coefficients, *p*: corresponding *p*-values. * *p* < 0.05.

## Data Availability

The sequence data have been successfully uploaded to the NCBI Sequence Read Archive (SRA). The submission has been reviewed and approved, and the reference number PRJNA1270052 has been assigned.

## Supplementary Materials

The following supporting information can be downloaded at: https://www.mdpi.com/article/10.3390/microorganisms13061317/s1, Table S1: Comparison of Vaginal Symptoms Over Three Months Among Post-menopause and Pre-menopause; Table S2: Comparison of Presence and Absence of Aerobic Vaginitis; Table S3: Comparison of Vaginal Symptoms Over Three Months Among Post-menopause and Pre-menopause with Different Pathogens; Table S4: Symptoms and CST classification among Cases without Pathogens.
