# Supplementary material for: The Vaginal Microbiome: Associations with Vaginal pH, Menopause and Metabolic Parameters
Source: Microorganisms. 2025 Jun 5;13(6):1317. doi: 10.3390/microorganisms13061317 (PMC12195170; doi:10.3390/microorganisms13061317)
Supplement: Supplementary file 1 [file microorganisms-13-01317-s001.zip › microorganisms-3630996-supplementary.pdf]

Table S1. Comparison of Vaginal Symptoms Over Three Months Among Post-menopause and Pre-menopause: (n=40).

	ALL			Post-menopause			Pre-menopause		
	No n/(%)	Yes n/(%)	*p*	No n/(%)	Yes n/(%)	*p*	No n/(%)	Yes n/(%)	*p*
CST [a]			.672			.551			.687
I	4(57.1)	3(42.9)		2(50.0)	2(50.0)		2(66.7)	1(33.3)	
II	1(100.0)	0(0.0)		1(100.0)	0(0.0)		-	-	
III	4(40.0)	6(60.0)		0(0.0)	1(100.0)		4(44.4)	5(55.6)	
IV	11(50.0)	11(50.0)		6(42.9)	8(57.1)		5(62.5)	3(37.5)	
Pathogen [b]			.605			-			.285
No	1(25.0)	3(75.0)		-	-		1(25.0)	3(75.0)	
Yes	19(52.8)	17(47.2)		9(45.0)	11(55.0)		10(62.5)	6(37.5)	
AV/BV(n=36) [b]			.717			.642			.234
BV	6(60.0)	4(40.0)		2(33.3)	4(66.7)		4(100.0)	0(0.0)	
AV	13(50.0)	13(50.0)		7(50.0)	7(50.0)		6(50.0)	6(50.0)	

CST: Community State Type

[a] Chi-square test、[b] Fisher’s exact test

AV：Aerobic Vaginitis、BV：Bacterial Vaginosis

Table S2. Comparison of Presence and Absence of Aerobic Vaginitis (n=36).

	ALL			Post-Menopause			Pre-Menopause		
	No n/(%)	Yes n/(%)	*p*	No n/(%)	Yes n/(%)	*p*	No n/(%)	Yes n/(%)	*p*
CST [a]			.319			.094			.565
I	1(16.7)	5(83.3)		0(0.0)	4(100.0)		1(50.0)	1(50.0)	
II	1(100.0)	0(0.0)		1(100.0)	0(0.0)		-	-	
III	3(37.5)	5(62.5)		1(100.0)	0(0.0)		2(28.6)	5(71.4)	
IV	5(23.8)	16(76.2)		4(28.6)	10(71.4)		1(14.3)	6(85.7)	

[a] Chi-square test

Table S3. Comparison of Vaginal Symptoms Over Three Months Among Post-menopause and Pre-menopause with Different Pathogens: (n=40).

Pathogen	ALL			Post-menopause			Pre-menopause		
	No n/(%)	Yes n/(%)	*p* [a]	No n/(%)	Yes n/(%)	*p* [a]	No n/(%)	Yes n/(%)	*p* [a]
Acinetobacter	1(100.0)	0(0.0)	1.00	1(100.0)	0(0.0)	.45	-	-	
Actinomyces	2(33.3)	4(66.7)	.661	2(50.0)	2(50.0)	1.00	0(0)	2(100)	.189
Atopobium	0(0)	1(100)	1.00	0(0)	1(100)	1.00	-	-	-
Anaerococcus	7(50.0)	7(50.0)	1.00	4(44.4)	5(55.6)	1.00	3(60.0)	2(40.0)	1.00
Bacteroides	3(42.9)	4(57.1)	1.00	2(40.0)	3(60.0)	1.00	1(50.0)	1(50.0)	1.00
Clostridium	0(0)	2(100)	.487	0(0)	2(100)	.479	-	-	-
Dialister	3(42.9)	4(57.1)	1.00	2(40.0)	3(60.0)	1.00	1(50.0)	1(50.0)	1.00
Escherichia	0(0)	2(100)	.487	1(100.0)	0(0.0)	.450	1(100.0)	0(0.0)	1.00
Finegoldia	4(36.4)	7(63.6)	.480	3(37.5)	5(62.5)	.670	1(33.3)	2(66.7)	.566
Fusobacterium	3(33.3)	6(66.7)	.451	3(42.9)	4(57.1)	1.00	0(0)	2(100)	.189
Gardnerella	10(58.8)	7(41.2)	.523	6(54.5)	5(45.5)	.406	4(66.7)	2(33.3)	.642
Gemella	1(12.5)	7(87.5)	.044*	1(16.7)	5(83.3)	.157	0(0)	2(100)	.189
Leptotrichia	2(50.0)	2(50.0)	1.00	2(66.7)	1(33.3)	.566	0(0)	1(100)	.450
Mycoplasma	1(50.0)	1(50.0)	1.00	1(50.0)	1(50.0)	1.00	-	-	-
Megasphaera	0(0)	2(100)	.450	0(0)	1(100)	1.00	0(0)	1(100)	1.00
Mobiluncus	3(75)	1(25)	.605	2(66.7)	1(33.3)	.566	1(100.0)	0(0.0)	1.00
Porphyromonas	7(53.8)	6(46.2)	1.00	6(60.0)	4(40.0)	.370	1(33.3)	2(66.7)	.566
Parvimonas	1(33.3)	2(66.7)	1.00	1(50.0)	1(50.0)	1.00	0(0)	1(100)	.450
Peptostreptococcus	1(50.0)	1(50.0)	1.00	1(50.0)	1(50.0)	1.00	-	-	
Peptococcus	1(50.0)	1(50.0)	1.00	1(50.0)	1(50.0)	1.00	-	-	
Peptoniphilus	6(50.0)	6(50.0)	1.00	4(40.0)	6(60.0)	1.00	2(100)	0(0)	.479
Prevotella	9(45.0)	11(55.0)	.752	6(46.2)	7(53.8)	1.00	3(42.9)	4(57.1)	.642
Sneathia	0(0)	1(100)	1.00	0(0)	1(100)	1.00	-	-	
Shuttleworthia	0(0)	1(100)	1.00	0(0)	1(100)	1.00	-	-	
Streptococcus	12(48.0)	13(52.0)	1.00	6(46.2)	7(53.8)	1.00	6(50.0)	6(50.0)	.670
Ureaplasma	8(72.7)	3(27.3)	.155	5(71.4)	2(28.6)	.160	3(75.0)	1(25.0)	.591

Fisher's exact test

Table S4. Symptoms and CST classification among Cases without Pathogens.

case #	Pathogen	Symptoms	CST
#7	negative	positive	III
#9	negative	negative	I
#18	negative	positive	IV
#37	negative	positive	III

CST: Community State Type
